# Rational quantum secret sharing

**DOI:** 10.1038/s41598-018-29051-z

**Published:** 2018-07-24

**Authors:** Huawang Qin, Wallace K. S. Tang, Raylin Tso

**Affiliations:** 1 School of Automatization, Nanjing University of Science and Technology, Nanjing, 210094 China; 2 Department of Electronic Engineering, City University of Hong Kong, Kowloon Tong, Hong Kong; 3 Department of Computer Science, National Chengchi University, Taipei, 11605 Taiwan

## Abstract

The traditional quantum secret sharing does not succeed in the presence of rational participants. A rational participant’s motivation is to maximize his utility, and will try to get the secret alone. Therefore, in the reconstruction, no rational participant will send his share to others. To tackle with this problem, we propose a rational quantum secret sharing scheme in this paper. We adopt the game theory to analyze the behavior of rational participants and design a protocol to prevent them from deviating from the protocol. As proved, the rational participants can gain their maximal utilities when they perform the protocol faithfully, and the Nash equilibrium of the protocol is achieved. Compared to the traditional quantum secret sharing schemes, our scheme is fairer and more robust in practice.

## Introduction

“Secret sharing” (SS) was first proposed by Shamir^1^, suggesting a secure way to distribute information (secret) to a set of participants. SS splits the secret into several parts and distributes them to different participants, so that only qualified participants can recover the original secret. In Shamir’s scheme, a participant is classified as “good” or “bad”. A good participant always performs the protocol faithfully, while the bad one would try his best to break it. However, this kind of classification may not reflect practical situations. Indeed, a participant can be neither good nor bad, but rational and always try to maximize his utility. Hence, each rational participant aims to get the secret, but at the same time, prevents others to get it.

The involvement of rational participants leads to a major problem in SS. In SS, a participant can recover the secret alone even not sending his share to others, if others have sent out theirs. On the other hand, if participants did not send their shares, none can recover the secret. Therefore, from the viewpoint of a rational participant, not sending his share weakly dominates sending his share. This implies the Nash equilibrium corresponds to the case that nobody sends his share to others, resulting in a failure of Shamir’s scheme in the presence of rational participants.

To mitigate this problem, Halpern *et al*.^2^ introduced the concept of “rational secret sharing” (RSS), and it has become an active area of research in recent years^3–5^. In classical RSS, signed share is used to prevent cheating of participants, while another approach is to use verifiable secret sharing^6^.

On the other hand, Hillery *et al*.^7^ have proposed “Quantum secret sharing” (QSS), which can be considered as an extension of Shamir’s SS into the area of quantum. In QSS, the secret is split, distributed and reconstructed by quantum operations. QSS provides more perfect security based on the quantum theory such as uncertainty principle and no-cloning theorem. Similar to SS schemes, the existing QSS schemes^8–21^ do not consider the rational behavior of participants. However, it is natural for the last participant, if he is rational, to generate the secret and quit with it alone. Thus, rational participants in QSS would always prefer not to provide their shares, making the conventional QSS schemes fail.

It should be emphasized those approaches suggested in RSS, such as signed share or verifiable SS, are based on unproven assumptions such as the intractability of integer factorization. In the quantum domain, participants and adversaries are always assumed to have unbounded computational power. As a result, these methods are inadequate for the design of rational quantum secret sharing (RQSS). In addition, there are other technical hurdles to be overcome for the design of RQSS, for example, the existing quantum signature schemes^22,23^ fail to deal with the entanglement among distributed shares, and a participant cannot generate copies of his share due to the no-cloning theorem.

Designing a workable RQSS is challenging but valuable, and it is also the main objective of this paper. In our proposal, the shared secret is assumed to be a *d*-dimensional quantum state. Some basic quantum operations, such as the quantum Fourier transform and quantum-controlled-not, are employed. Unlike our previous work^24^ and other QSS schemes, here the issue of “rationality” is focused. Game theory is introduced to analyze the rational behavior of participants, based on the concepts of rationality, fairness and Nash equilibrium. As with most of the QSS schemes^7–12^, the threshold structure of our scheme is (*n*, *n*) structure, meaning that all the *n* participants compose the only qualified set and any subset with fewer than *n* participants is a forbidden set. Our design can avoid rational participants to deviate from the protocol since an unfaithful act will not gain higher utility than a faithful one. As a result, the achieved Nash equilibrium corresponds to the case when all the rational participants perform the protocol faithfully, and eventually, the shared quantum state can be recovered with the involvement of all participants.

The rest of this paper is organized as follows. In Section 2, some correlative preliminaries are introduced. Section 3 describes the design of RQSS in details, while an example is provided in Section 4 to better illustrate the protocol. Section 5 proves the security of the proposal and Section 6 analyzes its Nash equilibrium. Section 7 compares the proposed scheme with our previous work. Finally, Section 8 concludes this paper.

## Preliminaries

The preliminaries of underlying QSS have been introduced adequately in other existing schemes, and hence only the preliminaries of rational part are focused. They are formalized in terms of rationality, fairness and Nash equilibrium, while quantum operations to be used in this work are also introduced.

### Rationality

The rationality of RQSS is specified by the following conditions. Considering two different strategies *a* and *a*′,

- if *O*_*i*_(*a*) = 1 and *O*_*i*_(*a*′) = 0, then *u*_*i*_(*a*) > *u*_*i*_(*a*′);
- if *O*_*i*_(*a*) = *O*_*i*_(*a*′) and *O*_*j*_(*a*′) < *O*_*j*_(*a*), then *u*_*i*_(*a*) > *u*_*i*_(*a*′).

*O*_*i*_(⋅) is a binary function and *u*_*i*_(⋅) is utility function of *P*_*i*_. *O*_*i*_(*a*) = 1 indicates that the participant *P*_*i*_ can recover the secret by applying strategy *a*, and *O*_*i*_(⋅) = 0 means that he can’t.

### Fairness

The fairness of RQSS is specified by the following conditions. Letting ${a}_{i}^{\ast }$ be the suggested strategy of the protocol and *a*_*i*_ be other possible strategy for participant *P*_*i*_,

- $${u}_{1}({a}_{1}^{\ast })={u}_{2}({a}_{2}^{\ast })=\cdots ={u}_{n}({a}_{n}^{\ast });$$
- $${u}_{i}({a}_{i}^{\ast }) > {u}_{j}({a}_{j}).$$

### Nash equilibrium

A RQSS protocol should achieve the Nash equilibrium such that no participant has any incentive to deviate from the protocol. A suggested strategy is said to be in Nash equilibrium when there is no incentive for any participant to deviate from it, given that everyone else is following this strategy. Formally, it can be described as follows.

For an arbitrary participant *P*_*i*_, if ${u}_{i}({a}_{i}^{\ast }|{a}_{-i}^{\ast }) > {u}_{i}({a}_{i}|{a}_{-i}^{\ast })$, then the strategy group ${a}^{\ast }=({a}_{1}^{\ast },{a}_{2}^{\ast },\cdots ,{a}_{n}^{\ast })$ is the Nash equilibrium. Here, ${a}_{i}^{\ast }$ and ${a}_{-i}^{\ast }=({a}_{1}^{\ast },\cdots ,{a}_{i-1}^{\ast },{a}_{i+1}^{\ast },\cdots ,{a}_{n}^{\ast })$ are the suggested strategy for participant *P*_*i*_ and all other participants, respectively; and ${u}_{i}(\cdot |{a}_{-i}^{\ast })$ represents *P*_*i*_’s utility given that all other *P*_*j*_ (*j* ≠ *i*) follow the suggested strategy.

### Quantum operations

#### Quantum Fourier transform

For a *d*-dimensional quantum state |*j*〉, *j* ∈ {0, 1, …, *d* − 1}, the quantum Fourier transform is defined as

1 $$F(|j\rangle )=\frac{1}{\sqrt{d}}\sum _{k=0}^{d-1}{\omega }^{kj}|k\rangle $$

where $\omega ={e}^{\frac{2\pi i}{d}}$. The corresponding quantum inverse Fourier transform is then given by

2 $${F}^{-1}(|j\rangle )=\frac{1}{\sqrt{d}}\sum _{k=0}^{d-1}{\omega }^{-kj}|k\rangle $$

#### *d*-dimensional quantum-controlled-not

Consider two *d*-dimensional quantum states |*j*_1_〉 and |*j*_2_〉, the *d*-dimensional quantum-controlled-not operation is expressed by:

3 $${\rm{C}}{\rm{N}}{\rm{O}}{\rm{T}}(|{j}_{1}\rangle ,|{j}_{2}\rangle )=(|{j}_{1}\rangle ,|{j}_{1}+{j}_{2}\rangle )$$

where |*j*_1_〉 and |*j*_2_〉 are referred as the control particle and target particle, respectively; and “+” is defined as the adder modulo *d* hereinafter.

## Design of RQSS

In RQSS, similar to other SS or RSS, there is a dealer who would like to distribute a secret to a set of participants. However, there are some district features in RQSS.

### Random structure

The dealer needs multiple rounds to distribute the shared secret to the participants. In each round, the dealer distributes the real secret (the shared secret) with a probability of *γ*, otherwise, a test secret is sent. Participants can only know whether the reconstructed secret is a real one or not after the dealer reveals the truth.

### Post verification

Dishonest participant should be punished and hence the behavior of participants must be verified. However, the methods employed in classical RSS are inadequate for RQSS due to the unbounded computational power in quantum domain. Therefore, quantum operations are to be applied.

### Generation of multiple same quantum states

In QSS, when the share is an unknown state, a participant cannot generate copies of his share due to the no-cloning theorem. If only one share is kept by a participant, only one secret can be reconstructed. Consequently, the participant who holds the reconstructed secret will have the privilege, breaking the fairness of RQSS. In order to resolve this problem, the dealer has to generate multiple same states and distribute to the participants, allowing all the qualified participants to get the reconstructed secret.

### Parameters setting based on Nash equilibrium

The parameters of RQSS should be properly set to guarantee that each honest participant can gain his maximal utility under the suggested strategy. The Nash equilibrium is to be achieved to ensure that the protocol can be performed robustly in the presence of rational participants.

The details of the RQSS protocol are given as follows. For the sake of clarity, the dealer and the *n* rational participants are referred as Alice and {Bob_1_, Bob_2_, …, Bob_*n*_}, respectively. The shared secret is assumed to be a *d*-dimensional quantum state, defined as $\phi =\sum _{j=0}^{d-1}{\alpha }_{j}|j\rangle$, where *α*_*j*_ are complex amplitudes and $\sum _{j=0}^{d-1}{|{\alpha }_{j}|}^{2}=1$.

To share *φ* among the *n* rational participants, Alice performs the following procedures for each round.

1. A specific coin having a probability of *γ* to be “1” (head) is tossed. If it is “1”, Alice generates *n* same real quantum states; otherwise, she generates *n* same test quantum states. For convenience, every one of these *n* quantum states is denoted as *φ*.
2. For each *φ*, the quantum inverse Fourier transform is applied to obtain *φ*′. For each *φ*′, Alice generates (*n* − 1) single particles, *p*_*i*_ = |*d* − 1〉 where *i* = 1, 2, …, (*n* − 1), and then performs *d*-dimensional quantum-controlled-not operation onto *φ*′ and each *p*_*i*_ in turns, where *φ*′ and *p*_*i*_ are the control particle and the target particle, respectively. An *n*-particle entangled state *Φ* is then resulted. Finally, Alice performs the quantum Fourier transform on each particle of *Φ* to obtain *Φ*′.
3. For every *Φ*′, Alice sends one particle of *Φ*′ to one participant sequentially. The particles transmission is protected by decoy particles, which are randomly selected from two bases, namely the *Z*-basis and the *X*-basis, as given in the following forms:
  $$Z=\{|j\rangle ,j=0,1,\ldots ,d-1\}$$
  and
  $$X=\{|{J}_{j}\rangle ,j=0,1,\ldots ,d-1\}$$
  where
  $$|{J}_{j}\rangle =\frac{1}{\sqrt{d}}\sum _{k=0}^{d-1}{\omega }^{kj}\,|k\rangle $$
4. For reconstruction, all the particles of one *Φ*′ will be sent to one of the participants, and eventually everyone will get one *Φ*′. The participant will perform the quantum inverse Fourier transform on every particle of his own *Φ*′ and get back *Φ*. By performing *d*-dimensional quantum-controlled-not operations, the quantum state *φ*′ and the (*n* − 1) single particles {*p*_1_, *p*_2_, …, *p*_*n*−1_} can be separated from *Φ*. Then the original state *φ* can be obtained by applying quantum Fourier transform onto *φ*′. For an arbitrary participant Bob_*i*_, the particles {*p*_1_, *p*_2_, …, *p*_*n*−1_} come from other (*n* − 1) participants. If other participants perform the protocol faithfully, then the obtained particles {*p*_1_, *p*_2_, …, *p*_*n*−1_} should all be in the state |*d* − 1〉. Therefore, by measuring these particles, Bob_*i*_ can deduce whether the corresponding participant has sent the correct particle or not. However, it should also be remarked that, there is still a probability of $\frac{1}{d}$ that Bob_*i*_ will get the correct measurement result even if the received particle is incorrect.
5. If a participant Bob_*i*_ finds that he did not receive any particle from Bob_*j*_ or the particle is not a correct one, Bob_*i*_ will publicize the cheating behavior of Bob_*j*_. Other participants will then terminate the protocol. When the protocol is terminated by any of the participants, the dealer will not continue the next round. As a result, participants will not be able to get the secret if the current round is not the real one.
6. If no cheating behavior is found, the dealer will reveal whether the secret in this round is the real secret or a test one. If it is the real secret, the protocol will be over. Otherwise, the dealer will start the next round.

## Example

To better illustrate the RQSS protocol, we consider a simple case with a dealer sharing a 3-dimensional quantum state to three participants.

In each round, Alice firstly decides to distribute the real secret or a test secret, *φ*, according to the result of coin tossing. Three same quantum states are then generated, each specified by *φ* = *α*_0_|0〉 + *α*_1_|1〉 + *α*_2_|2〉. By performing the quantum inverse Fourier transform as given in (2), Alice obtains

4 $$\phi ^{\prime} =\frac{1}{\sqrt{3}}[({\alpha }_{0}+{\alpha }_{1}+{\alpha }_{2})|0\rangle +({\alpha }_{0}+{\omega }^{2}{\alpha }_{1}+\omega {\alpha }_{2})|1\rangle +({\alpha }_{0}+\omega {\alpha }_{1}+{\omega }^{2}{\alpha }_{2})|2\rangle ]$$

Following the protocol, Alice generates two single particles, i.e. {*p*_1_ = |2〉, *p*_2_ = |2〉}. By applying 3-dimensional quantum-controlled-not operations onto *φ*′ and each *p*_*i*_ in turns, it results in the following quantum state

5 $${\Phi }=\frac{1}{\sqrt{3}}[({\alpha }_{0}+{\alpha }_{1}+{\alpha }_{2})|022\rangle +({\alpha }_{0}+{\omega }^{2}{\alpha }_{1}+\omega {\alpha }_{2})|100\rangle +({\alpha }_{0}+\omega {\alpha }_{1}+{\omega }^{2}{\alpha }_{2})|211\rangle ]$$

The quantum Fourier transform is then performed for each particle of *Φ* and finally Alice obtains

6 $$\begin{array}{rcl}{\Phi }^{\prime}  & = & \frac{1}{3}({\alpha }_{0}|000\rangle +{\omega }^{2}{\alpha }_{1}|001\rangle +\omega {\alpha }_{2}|002\rangle +{\omega }^{2}{\alpha }_{1}|010\rangle +\omega {\alpha }_{2}|011\rangle +{\alpha }_{0}|012\rangle \\  &  & +\,w{a}_{2}|020\rangle +{a}_{0}|021\rangle +{w}^{2}{a}_{1}|022\rangle +{a}_{1}|100\rangle +{w}^{2}{a}_{2}|101\rangle +w{a}_{0}|102\rangle \\  &  & +\,{w}^{2}{a}_{2}|110\rangle +w{a}_{0}|111\rangle +{a}_{1}|112\rangle +w{a}_{0}|120\rangle +{a}_{1}|121\rangle +{w}^{2}{a}_{2}|122\rangle \\  &  & +\,{a}_{2}|200\rangle +{w}^{2}{a}_{0}|201\rangle +w{a}_{1}|202\rangle +{w}^{2}{a}_{0}|210\rangle +w{a}_{1}|211\rangle +{a}_{2}|212\rangle \\  &  & +\,w{a}_{1}|220\rangle +{a}_{2}|221\rangle +{w}^{2}{a}_{0}|222\rangle )\end{array}$$

Based on the above operations, Alice will get three same entangled states and for simplicity, each one of them is denoted as *Φ*′. The three particles of each *Φ*′ will be sent to Bob_1_, Bob_2_ and Bob_3_, respectively. Consequently, each participant will have three particles which belong to one of the three entangled states.

In the reconstruction, the three particles of one *Φ*′ will be sent to one participant, and every participant will get one *Φ*′. When *Φ*′ is available, Bob_*i*_ will recover the original state *φ* by following Step (4) of the procedures as described in the last section. First, he gets back *Φ* by applying the quantum inverse Fourier transform on every particle of his *Φ*′. Then, two quantum-controlled-not operations are performed to separate the state *φ*′ and two single particles {*p*_1_, *p*_2_} from *Φ*. Finally, the original state *φ* = *α*_0_|0〉 + *α*_1_|1〉 + *α*_2_|2〉 is obtained by applying the quantum Fourier transform onto *φ*′. Bob_*i*_ can verify the honesty of other two participants by measuring {*p*_1_, *p*_2_}. If they sent Bob_*i*_ the correct particles, {*p*_1_, *p*_2_} should both be in the state |2〉.

If all the participants are honest, Alice will reveal whether the secret is a real one or not. If it is the real secret, the protocol will be over and all the participants obtain the secret. Otherwise, Alice will start again for the next round.

## Security analysis

In this section, the security of the proposed RQSS protocol is analyzed.

### Confidentiality

Given that the initial state Alice generated is $\phi =\sum _{j=0}^{d-1}{\alpha }_{j}|j\rangle ,$ by applying the quantum inverse Fourier transform onto *φ*, one has

7 $$\phi ^{\prime} =\sum _{j=0}^{d-1}{\alpha }_{j}(\frac{1}{\sqrt{d}}\sum _{k=0}^{d-1}{\omega }^{-kj}|k\rangle )=\frac{1}{\sqrt{d}}\sum _{k=0}^{d-1}(\sum _{j=0}^{d-1}{\alpha }_{j}{\omega }^{-kj}|k\rangle )$$

Then, with the (*n* − 1) quantum-controlled-not operations, *Φ* is obtained as follows1

Finally, after the *n* quantum Fourier transforms, one obtains

9 $$\begin{array}{lcl}{\Phi }^{\prime}  & = & \frac{1}{\sqrt{d}}\sum _{k=0}^{d-1}\{\sum _{j=0}^{d-1}{\alpha }_{j}{\omega }^{-kj}[(\frac{1}{\sqrt{d}}\sum _{{k}_{1}=0}^{d-1}{\omega }^{{k}_{1}k}|{k}_{1}\rangle )\\  &  & \times (\frac{1}{\sqrt{d}}\sum _{{k}_{2}=0}^{d-1}{\omega }^{{k}_{2}(k+d-1)}|{k}_{2}\rangle )\cdots (\frac{1}{\sqrt{d}}\sum _{{k}_{n}=0}^{d-1}{\omega }^{{k}_{n}(k+d-1)}|{k}_{n}\rangle )]\}\\  & = & \frac{1}{{(\sqrt{d})}^{n+1}}\sum _{k=0}^{d-1}[\sum _{j=0}^{d-1}{\alpha }_{j}{\omega }^{-kj}(\sum _{{k}_{1},\cdots ,{k}_{n}\in \{0,1,\cdots ,d-1\}}{\omega }^{{k}_{1}k+{k}_{2}(k+d-1)+\cdots +{k}_{n}(k+d-1)}|{k}_{1}{k}_{2}\cdots {k}_{n}\rangle )]\\  & = & \frac{1}{{(\sqrt{d})}^{n+1}}\sum _{k=0}^{d-1}[\sum _{j=0}^{d-1}{\alpha }_{j}{\omega }^{-kj}(\sum _{{k}_{1},\cdots ,{k}_{n}\in \{0,1,\cdots ,d-1\}}{\omega }^{({k}_{1}+\cdots +{k}_{n})k+({k}_{2}+\cdots +{k}_{n})(d-1)}|{k}_{1}{k}_{2}\cdots {k}_{n}\rangle )]\\  & = & \frac{1}{{(\sqrt{d})}^{n+1}}\sum _{k=0}^{d-1}[\sum _{{k}_{1},\cdots ,{k}_{n}\in \{0,1,\cdots ,d-1\}}(\sum _{j=0}^{d-1}{\alpha }_{j}{\omega }^{-kj+({k}_{1}+\cdots +{k}_{n})k+({k}_{2}+\cdots +{k}_{n})(d-1)}|{k}_{1}{k}_{2}\cdots {k}_{n}\rangle )]\end{array}$$

Since $\omega ={e}^{\frac{2\pi i}{d}}$ and *ω*^*d*^ = 1, one has $\,{\omega }^{({k}_{2}+\cdots +{k}_{n})(d-1)}=\,{\omega }^{d({k}_{2}+\cdots +{k}_{n})-({k}_{2}+\cdots +{k}_{n})}=\,{\omega }^{-({k}_{2}+\cdots +{k}_{n})}$. Therefore,

10 $$\begin{array}{rcl}{\Phi }^{\prime}  & = & \frac{1}{{(\sqrt{d})}^{n+1}}\sum _{k=0}^{d-1}[\sum _{{k}_{1},\cdots ,{k}_{n}\in \{0,1,\cdots ,d-1\}}({\omega }^{-({k}_{2}+\cdots +{k}_{n})}\sum _{j=0}^{d-1}{\alpha }_{j}{\omega }^{({k}_{1}+\cdots +{k}_{n}-j)k}|{k}_{1}{k}_{2}\cdots {k}_{n}\rangle )]\\  & = & \frac{1}{{(\sqrt{d})}^{n+1}}\sum _{j=0}^{d-1}[\sum _{{k}_{1},\cdots ,{k}_{n}\in \{0,1,\cdots ,d-1\}}({\omega }^{-({k}_{2}+\cdots +{k}_{n})}\sum _{k=0}^{d-1}{\alpha }_{j}{\omega }^{({k}_{1}+\cdots +{k}_{n}-j)k}|{k}_{1}{k}_{2}\cdots {k}_{n}\rangle )]\end{array}$$

Since $$\sum _{k=0}^{d-1}{\omega }^{k}=0,\,$$if $$j\ne \sum _{i=1}^{n}{k}_{i},\,$$then $$\sum _{k=0}^{d-1}{\alpha }_{j}{\omega }^{({k}_{1}+{k}_{2}+\cdots +{k}_{n}-j)k}=0.\,$$Otherwise, $$\sum _{k=0}^{d-1}{\alpha }_{j}{\omega }^{({k}_{1}+{k}_{2}+\cdots +{k}_{n}-j)k}=$$$$\sum _{k=0}^{d-1}{\alpha }_{j}=d{\alpha }_{j}$$. Therefore, only the item whose coefficient *α*_*j*_ with $$j=\sum _{i=1}^{n}{k}_{i}$$ in (10) can be retained, while other items will be disappeared. Therefore, the quantum state *Φ*′ can be simplified as

11 $$\Phi ^{\prime} =\frac{1}{{(\sqrt{d})}^{n-1}}\sum _{{k}_{1},\,{k}_{2},\cdots ,{k}_{n}\in \{0,1,\cdots ,d-1\}}{\omega }^{-({k}_{2}+\cdots +{k}_{n})}{\alpha }_{{k}_{1}+{k}_{2}+\cdots +{k}_{n}}|{k}_{1}{k}_{2}\cdots {k}_{n}\rangle $$

From (11), we can see that *Φ*′ is a symmetrical superposition state. Its particles can be in any state from {|0〉,|1〉, …, |*d* − 1〉} with the same probability equal to $\frac{1}{d}\sum _{j=0}^{d-1}{|{\alpha }_{j}|}^{2}=\frac{1}{d}$. It means that, if a participant measures his share, he will get a state from {|0〉, |1〉, …, |*d* − 1〉} with the same probability. The Von Neumann entropy of the share would approach its maximum, i.e. $S=-\,\frac{1}{d}\sum _{j=0}^{d-1}{\mathrm{log}}_{2}(\frac{1}{d})={\mathrm{log}}_{2}d$, implying that the quantum state of shares is independent of that of the quantum secret. Therefore, participants cannot get any information of the quantum secret from their own shares, and our scheme can meet the confidentiality^25^.

### Security for outside eavesdropping

In our scheme, the transmission of particles is protected by decoy particles. The decoy particles are randomly selected from the *Z*-basis or the *X*-basis, and the secret particle is randomly inserted into the decoy particles.

Since an attacker does not know the positions and bases of the decoy particles, if he intends to steal information by measuring the secret particle, he will probably measure the decoy particles with a random basis and would bring errors into the decoy particles. The probability of selecting a wrong basis for a decoy particle is $\frac{1}{2}$ and the participant has a probability of $\frac{d-1}{d}$ to obtain a wrong value for the decoy particle. Therefore, the error rate of one decoy particle for eavesdropping is $\frac{d-1}{2d}$^26^. If there are *l* decoy particles, eavesdropping can be detected with a probability of $1-{(\frac{d+1}{2d})}^{l}$. When *l *is sufficiently large, the probability will be close to 1.

Besides direct eavesdropping, another famous attack from outsider is known as “entangle-and-measure”. The attacker will entangle an ancillary particle on the secret particle, and then measure the ancillary particle to steal information. It is remarked that, according to the results in^26^, this attack can also be detected due to the errors of decoy particles.

### Security for dishonest participant

In our scheme, the secret state *φ* is hidden in the entangled state *Φ*′ as given in (11). As described in Section 5.1, *Φ*′ is a symmetrical superposition state and each of its particles can be in any state from {|0〉, |1〉, …, |*d* − 1} with the same probability. Even if (*n* − 1) participants work together, it is still impossible for them to get the initial secret state.

Without loss of generality, we assume {Bob_2_, Bob_3_, …, Bob_*n*_} measure their particles and obtain results {*r*_2_, *r*_3_, …, *r*_*n*_}. Bob_1_’s particle will become the following state

12 $$\psi =\sum _{j=0}^{d-1}{\omega }^{-({r}_{2}+\cdots +{r}_{n})}{\alpha }_{j+{r}_{2}+\cdots +{r}_{n}}|j\rangle $$

From (12), we can see that {Bob_2_, Bob_3_, …, Bob_*n*_} still cannot get the secret state *φ* without Bob_1_. This confirms that the secret state can be recovered only if all participants are available, and hence collusion attack from dishonest participants will not succeed.

## Nash equilibrium

In our scheme, there are four possible strategies when a rational participant performs the protocol.

- *a*_1_: send the correct particles to other participants;
- *a*_2_: remain silent, i.e., not send any particles to other participants;
- *a*_3_: send the forged particles to other participants;
- *a*_4_: measure the particles and then send them to other participants, i.e., the shared state will be destroyed.

The participant may have the following four utilities.

- *U*_1_: he gets the secret but the other participants do not;
- *U*_2_: he gets the secret and same for the other participants;
- *U*_3_: he does not get the secret and neither the other participants;
- *U*_4_: he does not get the secret but the other participants get the secret.

For a rational participant, it is obvious that *U*_1_ > *U*_2_ > *U*_3_ > *U*_4_.

Now, we analyze the utility of an arbitrary participant, Bob_*i*_, performing different strategies in a round *j*.

1. Perform strategy *a*_1_: his utility is *U*_2_.
2. Perform strategy *a*_2_: if the secret in this round is the real secret (the probability is *γ*), his utility is *U*_1_; otherwise, his utility is *U*_3_. So the utility under *a*_2_ is *γU*_1_ + (1 − *γ*)*U*_3_.
3. Perform strategy *a*_3_: if the secret in this round is the real secret (the probability is *γ*), his utility is *U*_1_; otherwise, his utility is *αU*_3_ + (1 − *α*)*U*_2_, where *α* is the probability that his cheating behavior is detected by the others. As explained in Section 3, we have $\alpha =\frac{1}{d}$ in our scheme. Therefore, the utility under *a*_3_ is $\gamma {U}_{1}+(1-\gamma )[\frac{1}{d}{U}_{3}+(1-\frac{1}{d}){U}_{2}]$.
4. Perform strategy *a*_4_: it is similar to case (3) and the utility also equals to $\gamma {U}_{1}+(1-\gamma )[\frac{1}{d}{U}_{3}+(1-\frac{1}{d}){U}_{2}]$.

The utility of Bob_*i*_ under different strategy is summarized below:

- *u*_*i*_(*a*_1_) = *U*_2_
- *u*_*i*_(*a*_2_) = *γU*_1_ + (1 − *γ*)*U*_3_
- $${u}_{i}({a}_{3})=\gamma {U}_{1}+(1-\gamma )[\frac{1}{d}{U}_{3}+(1-\frac{1}{d}){U}_{2}]$$
- $${u}_{i}({a}_{4})=\gamma {U}_{1}+(1-\gamma )[\frac{1}{d}{U}_{3}+(1-\frac{1}{d}){U}_{2}]$$

Since *U*_2_ > *U*_3_, it can be easily deduced that *u*_*i*_(*a*_2_) is always less than *u*_*i*_(*a*_3_) or *u*_*i*_(*a*_4_). Now, letting ${U}_{2} > \gamma {U}_{1}+(1-\gamma )[\frac{1}{d}{U}_{3}+(1-\frac{1}{d}){U}_{2}]\,$or $\gamma  < \frac{{U}_{2}-{U}_{3}}{d{U}_{1}-(d-1){U}_{2}-{U}_{3}}$, the rational participant Bob_*i*_ will always choose *a*_1_ as his strategy since *u*_*i*_(*a*_1_) > *u*_*i*_(*a*_3_) = *u*_*i*_(*a*_4_) > *u*_*i*_(*a*_2_).

Therefore, if the parameter *γ* is set to satisfy the inequality condition $\gamma  < \frac{{U}_{2}-{U}_{3}}{d{U}_{1}-(d-1){U}_{2}-{U}_{3}}$, every rational participant will choose *a*_1_ as his optimal strategy, which is the Nash equilibrium, and perform the protocol faithfully.

## Comparison

In our scheme, it is assumed that the shared secret is a *d*-dimensional quantum state and quantum operations, such as the quantum Fourier transform and quantum-controlled-not, are employed. Although similar assumptions and operations are used in our previous work^24^, the design and focus of this paper are totally different. The main feature of our scheme is to manage the “rationality”. The scheme in^24^ is only a traditional QSS scheme without considering the “rationality”.

In particular, we introduce the game theory into the QSS to analyze the rational behavior of participant, based on respective definitions of rationality, fairness and Nash equilibrium. The proposed RQSS possesses some distinct features as discussed in Section 3, including the random structure, post verification based on quantum operation, and parameters setting based on Nash equilibrium. Furthermore, we analyze different strategies and utilities of the rational participant, and derive conditions to ensure rational participants to follow the protocol faithfully, by achieving the Nash equilibrium. All these novel contents do not appear in^24^.

Indeed, the protocol of RQSS is also different from that suggested in^24^. In our scheme, the dealer applies the quantum inverse Fourier transform onto the shared state, and then performs the quantum-controlled-not operations and quantum Fourier transform to hide the shared state into an entangled state. For reconstruction, participants need to perform reverse operations, including the quantum inverse Fourier transform, the quantum-controlled-not and the quantum Fourier transform, to obtain the shared state and the verification states. In contrast, participants under the scheme in^24^ only perform single-particle measurements and unitary operations to recover the shared state. Such a reconstruction process is not preferable, as participants cannot obtain the verification states to verify the faithfulness of other participants.

## Conclusion

In this paper, we have proposed a RQSS scheme to manage rational participants who try to maximize their utilities. By using quantum operations, the dealer encodes the secret state into an entangled state and distributes to the participants, while participants can use reverse operations to recover the secret state. The behavior of the rational participant is analyzed with the use of Game theory, and suitable mechanisms are proposed to motivate rational participants to perform the protocol faithfully. As proved, our scheme is fair and secure, and the suggested strategy achieves the Nash equilibrium. Compared to the existing QSS schemes, our scheme is more practical in the presence of rational participants.

The entangled state is indispensable in our scheme. Compared with the single-qubit state, the multi-particles entangled state is harder to be prepared with the current technologies. However, as discussed in^27–32^, some practical ways are possible to generate the entangled state. With the rapid development of quantum technology, generating entangled states would become easier in the future, making our scheme more practical.
